# Herpes Simplex Virus Type 1 Clinical Isolates Respond to UL29-Targeted siRNA Swarm Treatment Independent of Their Acyclovir Sensitivity

**DOI:** 10.3390/v12121434

**Published:** 2020-12-13

**Authors:** Kiira Kalke, Jenni Lehtinen, Jelena Gnjatovic, Liisa M. Lund, Marie C. Nyman, Henrik Paavilainen, Julius Orpana, Tuomas Lasanen, Fanny Frejborg, Alesia A. Levanova, Tytti Vuorinen, Minna M. Poranen, Veijo Hukkanen

**Affiliations:** 1 Institute of Biomedicine, University of Turku, 20520 Turku, Finland; jen_ni_90@hotmail.com (J.L.); jelena.j.gnjatovic@utu.fi (J.G.); liisa.lund@utu.fi (L.M.L.); marie.nyman@utu.fi (M.C.N.); hojpaa@utu.fi (H.P.); jamorp@utu.fi (J.O.); tuaula@utu.fi (T.L.); fanny.o.frejborg@utu.fi (F.F.); tytti.vuorinen@tyks.fi (T.V.); 2 Molecular and Integrative Biosciences Research Programme, Biological and Environmental Sciences, University of Helsinki, 00790 Helsinki, Finland; alesia.levanova@helsinki.fi (A.A.L.); minna.poranen@helsinki.fi (M.M.P.); 3 Clinical Microbiology, Turku University Hospital, 20520 Turku, Finland

**Keywords:** herpes simplex virus, antiviral, RNAi, siRNA, acyclovir, acyclovir resistance

## Abstract

Acyclovir is the drug of choice for the treatment of herpes simplex virus (HSV) infections. Acyclovir-resistant HSV strains may emerge, especially during long-term drug use, and subsequently cause difficult-to-treat exacerbations. Previously, we set up a novel treatment approach, based on enzymatically synthesized pools of siRNAs, or siRNA swarms. These swarms can cover kilobases-long target sequences, reducing the likelihood of resistance to treatment. Swarms targeting the *UL29* essential gene of HSV-1 have demonstrated high efficacy against HSV-1 in vitro and in vivo. Here, we assessed the antiviral potential of a UL29 siRNA swarm against circulating strains of HSV-1, in comparison with acyclovir. All circulating strains were sensitive to both antivirals, with the half-maximal inhibitory concentrations (IC_50_) in the range of 350–1911 nM for acyclovir and 0.5–3 nM for the UL29 siRNA swarm. Additionally, we showed that an acyclovir-resistant HSV-1, devoid of thymidine kinase, is highly sensitive to UL29 siRNA treatment (IC_50_ 1.0 nM; I_max_ 97%). Moreover, the detected minor variations in the RNAi target of the HSV strains had no effect on the potency or efficacy of UL29 siRNA swarm treatment. Our findings support the development of siRNA swarms for the treatment of HSV-1 infections, in order to circumvent any potential acyclovir resistance.

## 1. Introduction

Acyclovir (ACV) and other structurally related analogues of deoxyguanosine are well-established antivirals for treatment of herpes simplex virus (HSV) infection, with high specificity and low toxicity [1,2]. In certain recurrent herpes infections, prophylactic ACV treatment is needed to prevent potential exacerbations caused by the reactivation of HSV from its latent state, which the virus establishes after primary, lytic infection. However, if patients require such long-term prophylactic treatment, as is needed by those suffering from herpes keratitis, ACV-resistant HSV strains can emerge not only in immunocompromised, but also in immunocompetent individuals [3,4,5,6]. The ACV resistance of HSV-1 is mainly caused by mutations in the *UL23* gene encoding the HSV thymidine kinase [7,8]. The prevalent inter- and intrastrain genetic variability in *UL23* among circulating strains of HSV-1 [7] causes differences in ACV sensitivity between and among circulating clinical HSV-1 strains [6,9].

Small interfering RNA (siRNA) drugs are recognized as potential antivirals for treatment of virus infections [10]. Antiviral siRNAs are, however, challenged by their limited target sequence, usually covering only 20–25 base pairs (bp), making them susceptible to variations in viral genomes and to emergence of escape mutants. To enable better tolerance for genetic diversity and better treatment for medication-resistant viral infections, we have developed siRNA swarms, which are pools of enzymatically synthesized Dicer substrate siRNAs that can cover even several kilobases of the viral targets [11]. The individual Dicer substrate siRNA molecules in the swarm are 25 bp long, and upon introduction to the cells, are processed by the endogenous human Dicer, resulting in enhanced efficacy and potency [12,13]. Moreover, siRNA swarms may be directed against multiple viral genes simultaneously, when chimeric constructs are used as sources for the siRNA swarm synthesis [14]. Antiviral siRNA swarms have previously proven efficient against various viruses and viral strains [11,13,14]. Recently, siRNA swarms were developed for increased antiviral efficacy and plasma stability with incorporated modified nucleotides [15].

We have previously developed siRNA swarms against HSV-1 [13,15,16,17,18], which potentially provide novel options for therapy as topical treatments of herpes keratitis and other superficial infections. Of the studied HSV target genes, the essential *UL29* gene has proven most suitable [18]. The gene codes for the single stranded DNA-binding protein ICP8, which is required throughout the biphasic DNA replication of HSV-1 [19]. As a target for RNAi, the *UL29* gene has proven efficient for various recombinant, reference and clinical HSV-1 strains, both in vitro [18] and in vivo [16,20], with no emerging resistance over multiple passages in vitro [18]. 

Here, our aim is to assess the antiviral potential of the UL29 siRNA swarm against circulating HSV-1 strains and a thymidine kinase *(tk)* deficient HSV-1 [21], in comparison with ACV. We show that treatment with the UL29 siRNA swarm inhibits 98% or more of viral production of every studied circulating strain of HSV-1, and that all studied circulating strains were susceptible to the treatment with only minimal deviation in sensitivity. Furthermore, we show that the *tk-*deficient HSV-1 was susceptible to treatment with the UL29 siRNA swarm, suggesting the potential of UL29-targeted RNAi in treatment of ACV-resistant herpes infections.

## 2. Materials and Methods

### 2.1. Cells

Vero cells (CCL-81, ATCC, Manassas, VA, USA), used for all experiments, were maintained in M199 medium (Gibco, Waltham, MA, USA) with 5% fetal bovine serum (FBS) and gentamycin.

### 2.2. HSV-1 Strains

Seventeen anonymous archival HSV isolates were derived from clinical specimens sent to a local clinical virology diagnostic service (Virus Diagnostic Unit of the Turku University Hospital, research permit #J10/17). The isolates were confirmed as HSV-1 by immunoperoxidase rapid culture assay [22]. In addition to these clinical isolates, HSV-1 reference strains, 17+ and F, as well as an ACV-resistant, *tk*-deficient strain (HSV-1 (Δ305), [21]) were studied. For a summary of viruses used, please see Table 1. All viruses were propagated into supernatant stocks in Vero cells, as described previously [18]. The stocks of the circulating strains were of low passage (passage #1–4) and had viral titers varying from 3 × 10^5^ to 3 × 10^8^ plaque forming units (pfu) per mL.

### 2.3. Acyclovir Sensitivity

ACV sensitivity was studied by a plaque reduction assay on Vero cells [23]. For five of the virus isolates (F-14g, F-17, F-18g, M-15, M-19), as well as for HSV-1 (Δ305) and HSV-1 (17+), previously published data of acyclovir sensitivity [9] were used and were thus not repeated here in vitro. The assay was conducted on a 96-well plate in duplicates with infections of 100 pfu/well at four hours post treatment (hpt), and quantified three days post infection (dpi) by counting the plaques. The acyclovir concentrations ranged from 0.03 to 128 µg/mL, corresponding approximately to 0.1 and 570 µM, respectively. The upper limit of susceptibility was considered to be the half maximal inhibitory concentration (IC_50_) value of 1.90 μg/mL (8.43 μM). The reduction of viral growth (%) by ACV was determined against untreated, infected samples.

### 2.4. Sensitivity to RNAi with the UL29 siRNA Swarm 

The antiviral UL29-specific siRNA swarm, targeting a 653 bp sequence of the essential *UL29* gene, was synthesized as previously described [13,17,18]. The transfections of the siRNA swarm, at different concentrations of the siRNA, were performed in 40–60% confluent Vero cell cultures on 96-well plates using Lipofectamine RNAiMAX (#13778-150; Invitrogen, Carlsbad, CA, USA) according to the manufacturer’s protocol. The concentration of the siRNA swarm ranged from 0.4 to 100 nM, as specified in the Results section. Four hours post treatment (hpt), the cells were infected with 100 pfu of each of the viral strains, as previously described [18]. Our data represent at least five biological replicates per RNA concentration for each virus. At 3 dpi, the virus was quantified by titration of the culture supernatants on Vero cells using six parallel wells for each dilution. The inhibition of virus production by the anti-UL29 siRNA swarm was determined as a reduction of viral growth (%) in comparison with mock samples transfected with water.

### 2.5. Sequencing of the UL29 Swarm Target of the Strains

The UL29 siRNA swarm target area (nucleotides 59,954–59,302 as according to HSV-1 (17+) sequence, Genbank accession number JN555585.1) was sequenced from the strains of which there was yet no sequence data available. The sequencing of the strains was conducted by amplifying the target sequence from viral stocks using Phusion polymerase (Thermo Scientific, Waltham, MA, USA) and the primers CCTGCACGCTGGGGG and CAGTGCCACGGGGTGTTC. The products were analyzed and purified using agarose gel electrophoresis and submitted for sequencing (LightRun, Eurofins Genomics, Denmark). The siRNA swarm target sequences of the other strains were obtained from Genbank (Table 1). The Genbank accession numbers of the novel target sequences are MW287999-MW288011.

### 2.6. Data Analysis

The IC_50_ values and maximum inhibition rates (I_max_) were calculated using a nonlinear, sigmoidal fit in Origin version b9.3.2.303 (OriginLab Corporation, Northhampton, MA, USA). The correlation analyses were done using Spearman’s nonparametric correlation analysis in GraphPad Prism version 8.4.3. (GraphPad Software Inc., San Diego, CA, USA).

## 3. Results

Seventeen HSV-1 strains circulating in Finland (Table 1), two common reference strains, HSV-1 (17+) and HSV-1 (F), as well as a *tk*-deficient virus HSV-1 (Δ305) [21] were tested for sensitivity to treatment with ACV or a UL29 siRNA swarm. The antiviral assays determining the sensitivities were conducted on Vero cells with 100 pfu per well on a 96-well plate format. UL29 siRNA swarm-related toxicity was evaluated in Vero cells, and was minimal, remaining similar throughout the used siRNA concentration range (Figure S1).

**Figure 1:**
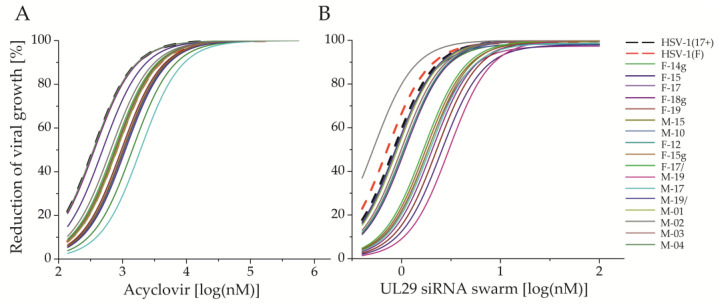
Inhibition of viral shedding in response to (**A**) acyclovir (ACV) or (**B**) UL29 siRNA swarm. The fitted curves represent circulating clinical (solid lines, N = 17) or reference strains (dashed lines, N = 2) of HSV-1. In all antiviral assays, Vero cells were treated with the antiviral agent on 96-well plates, infected 4 h post treatment (hpt) with 100 plaque forming units (pfu) per well, and finally quantified for reduction of viral production into the culture medium at 72 hpt. The dose is shown as log (nM) for both antivirals. The response is shown as inhibition percentage (%) in comparison to untreated cells for ACV and to water-treated cells for the UL29 siRNA swarm. The ACV plaque reduction assay was conducted in duplicates, and the antiviral siRNA swarm assay with five or more biological replicates for each concentration.

None of the studied circulating HSV-1 strains was resistant to ACV (Table 2) and all were dose-responsive to ACV treatment (Figure 1A). Their ACV sensitivity (half maximal inhibitory concentration, IC_50_) ranged from 349.7 to 1911.2 nM (0.08 to 0.43 µg/mL), with an average of 844.0 nM (standard deviation, SD 379.1 nM) (Table 2). All studied circulating and reference strains were susceptible to treatment with UL29 siRNA swarm (Table 2). The sensitivity (IC_50_) of circulating strains to UL29 siRNA swarm treatment ranged from 0.5 nM to 3.0 nM, with an average of 1.3 nM (SD 0.68 nM), whereas the reference strains HSV-1 (17+) and HSV-1 (F) had sensitivities of 0.8 and 0.7 nM, respectively (Table 2). All of the strains demonstrated dose-responsiveness to UL29 siRNA swarm treatment (Figure 1B). 

**Figure 2:**
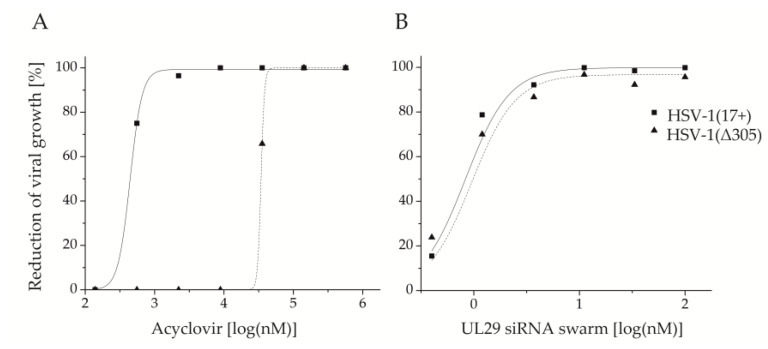
The thymidine kinase (*UL23*) defective HSV-1 strain (Δ305), and reduction of its growth in response to elevating concentrations of (**A**) acyclovir (ACV) or (**B**) UL29 siRNA swarm. HSV-1 (Δ305) is shown as a dotted line and triangles. The corresponding curve of the reference strain HSV-1 (17+) is shown with a solid line and squares. Both the UL29 siRNA swarm and ACV were studied for prophylactic efficacy, with treatment given four hours before infection. The assays were conducted on 96-well plates with a virus dose of 100 pfu per well. The dose is shown as log(nM) for both antivirals and the response is shown as inhibition percentage (%) versus untreated cells for ACV and versus water-treated for the UL29 siRNA swarm. The ACV plaque reduction assay was conducted in duplicates, and the antiviral siRNA swarm assay with five or more biological replicates for each concentration.

HSV-1 (Δ305) is ACV-resistant (IC_50_: 25,468.8 nM) due to the lack of a functional *tk* gene (Table 2, Figure 2A). However, HSV-1 (Δ305) was clearly susceptible to treatment with UL29 siRNA swarm (IC_50_: 1.0 nM) (Table 2), with similar dose-responsiveness to the reference strain HSV-1 (17+) (Figure 2B). The UL29 siRNA swarm treatment specificity in Vero cells was tested for both HSV-1 (Δ305) and HSV-1 (17+) using a nonspecific siRNA swarm (Figure S2). Even at the highest siRNA swarm concentration used for the antiviral assays (100 nM), treatment with the nonspecific siRNA swarm did not lead to any reduction of viral growth (in comparison with water treatment) with either of the viruses. 

**Figure 3:**
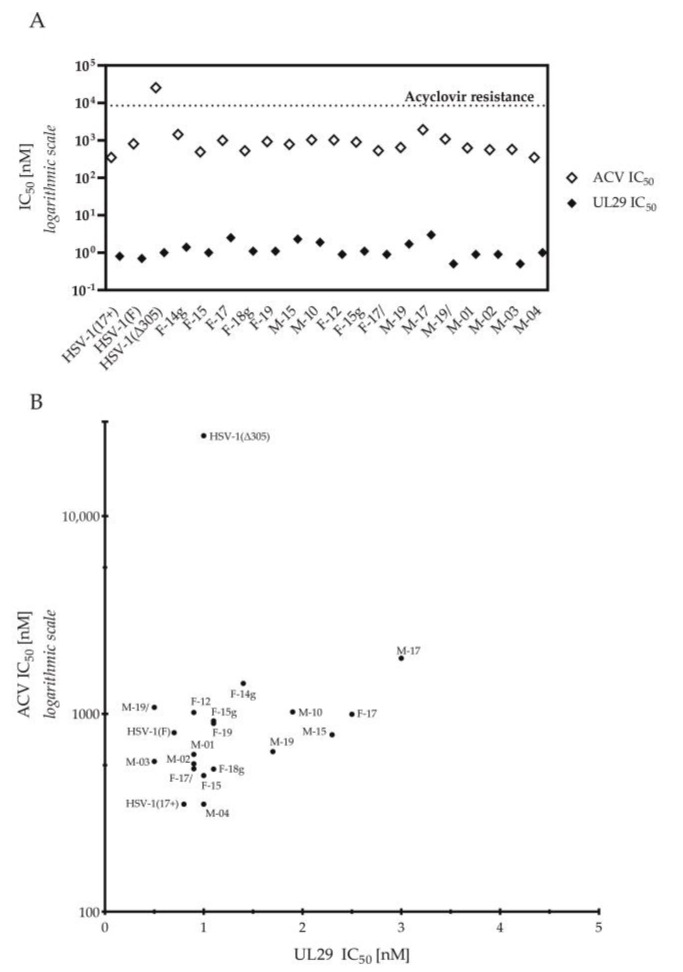
Comparison of the antiviral susceptibility of the strains. (**A**) Acyclovir (ACV) and UL29 RNAi sensitivities (IC_50_) of HSV-1 strains. The dotted line shows the limit of ACV resistance (1.9 µg/mL, 8.43 µM). (**B**) A scatter plot analysis for correlation of ACV and UL29 sensitivities. The sensitivity (IC_50_) of the HSV-1 strains for ACV is shown on the y-axis and for UL29 siRNA swarm shown on the x-axis. The values are shown as nM. ACV sensitivity is plotted in logarithmic scale. There was no significant correlation between the ACV sensitivities and UL29 siRNA swarm sensitivities of the circulating strains (*p* = 0.49) (Spearman’s *r* = 0.19).

Out of all tested viruses, only HSV-1 (Δ305) exceeded our limit of acyclovir resistance (Figure 3A), clearly deviating from the other viruses with a more than fifteen-fold higher ACV IC_50_ value (Figure 3B). Still, the sensitivity of HSV-1 (Δ305) to treatment with UL29 siRNA swarm was similar to other strains (Table 2, Figure 3). Altogether, the efficacies of ACV or the UL29 siRNA swarm varied only modestly among the circulating strains (Figure 1 and Figure 3A). Furthermore, the genital sample-derived HSV-1 strains, F14g, F18g, and F15g, were similar to the other circulating strains in both ACV and UL29 siRNA swarm sensitivity (Table 2, Figure 3) and in dose-response profiles (Figure 1). In comparison with other circulating strains, M-17 was less sensitive to both treatments (Figure 3B). Nevertheless, no correlation between the ACV sensitivities and UL29 siRNA swarm sensitivities of the circulating strains was detected (Figure 3B) (Spearman’s *r* = 0.19, *p* = 0.49, nonsignificant).

The UL29 siRNA swarm target sequence, which is based on the HSV-1 (17+) *UL29* gene sequence (JN555585.1; nucleotides 59,954–59,302), was determined for all strains of which there is not a Genbank accession available. A majority of the strains had over 99.7% similarity with the siRNA swarm target sequence, yet only M-19/had complete similarity with the HSV-1 (17+) UL29 target. F-18g, with 1.08 single nucleotide polymorphisms per 100 nucleotides in the UL29 siRNA swarm target area, differed the most (Table 3). The detected differences in the RNAi target sequence did not affect the UL29 swarm RNAi efficacy, as no correlation between the sequence similarity and sensitivity to the UL29 siRNA swarm or the maximal inhibitory efficacy of the UL29 siRNA swarm could be detected (Figure S3).

## 4. Discussion

In the current study, we evaluated the sensitivity of seventeen Finnish circulating strains of HSV-1 to both acyclovir and to RNAi with a UL29 siRNA swarm [13,15,16,17,18]. The target for our RNAi was the essential *UL29* gene, whereas the sensitivity of a given HSV strain to ACV is dependent on its thymidine kinase or DNA polymerase genes [2,6,7]. All the studied viruses, including the ACV-resistant *tk*-deficient HSV-1 (Δ305), proved to be susceptible to the UL29 siRNA swarm. 

Previously, we have shown the efficacy of the UL29 siRNA swarm in epithelial, retinal, and neuronal cell types [13,15,17,18]. Here, in order to compare the antiviral efficacies of the UL29 siRNA swarm and ACV, we utilized Vero cells for all antiviral assays, as they are commonly used for determining HSV-1 susceptibility to antivirals [9,23]. Furthermore, due to the minimal interferon response of Vero cells, they serve well in predicting the sequence-specific silencing of the viral replication in antiviral RNAi. We show that Vero cells are compatible with UL29-specific antiviral siRNA swarm treatment (Figure 1, Figure 2 and Figure 3), without any cytotoxicity (Figure S1) or nonspecific antiviral efficacy (Figure S2). Altogether, the detected antiviral efficacy of the UL29 siRNA swarm in Vero cells is similar to that previously detected in human epithelial cells [13,17,18], favoring the translationality of the results derived from Vero cells. 

For our RNAi target, we decided to exploit the *UL29* gene, instead of targeting the nonessential thymidine kinase (*UL23* gene). SiRNAs targeting genes, such as *UL23*, which are not essential for viral replication in many settings [21], would not serve as novel antivirals against HSV-1, and might even select for mutant viral strains. Moreover, as simultaneous substantial mutations in *UL29* and *UL23* have not been reported to our knowledge, the UL29-targeted antiviral RNAi therapy is a promising alternative for the treatment of HSV-1 infections with decreased response to ACV due to mutations in the *UL23* gene.

All seventeen circulating HSV-1 strains were susceptible and dose-responsive to ACV (Table 2, Figure 1A). The *tk*-deficient HSV-1(Δ305) was the only one of the studied viruses resistant to ACV (Table 2, Figure 2A and Figure 3A), as none of the circulating strains exceeded the limit of resistance (Figure 3A). Altogether, the ACV sensitivity of the strains was consistent with previous literature, and the ACV IC_50_ value of HSV-1(Δ305) was well in line with other published ACV-resistant strains [4,6,9].

The UL29 siRNA swarm led to the reduction of viral growth in a dose-responsive manner (Figure 1B), similarly to ACV. All reference and circulating strains were shown to be susceptible, with little to no deviation in the IC_50_ value (Table 2, Figure 1B and Figure 3). Moreover, the UL29 siRNA swarm treatment reduced the growth and shedding of all circulating strains by more than 98% (Figure 1B, Table 3) at well-tolerated concentrations (Figure S1).

Supporting our intent to develop siRNA swarms for treatment of ACV-resistant herpes, we observed the susceptibility of an ACV-resistant virus to UL29 siRNA swarm treatment (Figure 2B). The responsiveness to the treatment was similar to that of the reference strain HSV-1 (17+) (Figure 2B), which has served as the sequence template for the UL29 siRNA swarm [13]. As mutations in the *UL23* sequence are the most prevalent cause of ACV resistance in circulating strains of HSV-1 [7,8], the capability to treat *UL23*-deficient variants of HSV-1 with an siRNA swarm enhances the potential of the treatment approach.

Notably, M-17, the least sensitive circulating strain to both ACV and to UL29 siRNA swarm treatment (Figure 3B), was eliminated almost completely (99.8%) by UL29 siRNA swarm treatment (Table 3). Moreover, no significant correlation between ACV and UL29-targeted RNAi sensitivities was detected (Figure 3B), indicating that the efficacy of the UL29 siRNA swarm is independent of ACV sensitivity of circulating strains, as was confirmed by its potency and efficacy against the ACV-resistant HSV-1(Δ305) (Figure 2B, Figure S2). 

Additionally, we showed that the chosen target area of the UL29 siRNA swarm has minimal but existing diversity among the studied strains (Table 3). The differences in sequence, which ranged from 0.15 to 1.08 nucleotide differences per 100 nucleotides (Table 3), did not have an effect on the UL29 siRNA swarm efficacy or potency (Figure S3), consistent with the potential of the siRNA swarm approach to overcome viral escape mutants in therapeutic use.

In this proof-of-concept study, we provided evidence supporting the treatment of ACV-resistant HSV-1 infections with an siRNA swarm. Furthermore, we confirmed the compatibility of Vero cells with siRNA therapy, as well as the dose-responsivity of HSV-1 to the UL29 siRNA swarm. Collectively, our results support further development of siRNA swarm therapies for difficult-to-treat topical HSV infections, as all the tested circulating clinical HSV-1 strains were susceptible to UL29 siRNA swarm treatment with high efficacy and sensitivity, regardless of slight variations in target sequence. Our results indicate that UL29-targeted siRNA swarms can be used to treat ACV-resistant herpes infections with a low probability for emergence of escape mutants. Taken together, the UL29 siRNA swarm is highly promising for future treatment of HSV-1 infections, independent of the ACV susceptibility of the causative viral strain.

## Figures and Tables

**Table 1:** HSV-1 strains used in this study.

Strain Code	Sample No.	Sample	Gender	Genbank Accession ^1^
**HSV-1 (17+)**	-	-	-	JN555585.1
**HSV-1 (F)**	-	-	-	GU734771.1
**HSV-1 (Δ305)**	-	-	-	-
**F-14g**	H12114	genital	F	MH999844.1
**F-15**	H12115	-	F	-
**F-17**	H12117	blister	F	MH99845.1
**F-18g**	H12118	genital	F	MH99847.1
**F-19**	H12119	lip	F	-
**M-15**	H1215	blister	M	MH99846.1
**M-10**	H15110	-	M	-
**F-12**	H15112	blister	F	-
**F-15g**	H15115	genital	F	-
**F-17/**	H15117	facial	F	-
**M-19**	H15119	blister	M	MH999850.1
**M-17**	H1517	genital	M	-
**M-19/**	H1519	-	F	-
**M-01**	H151V1	blister	-	-
**M-02**	H151V2	blister	-	-
**M-03**	H151V3	blister	-	-
**M-04**	H151V4	blister	-	-

^1^ Indicated when the whole genome sequence is available.

**Table 2:** Finnish clinical HSV-1 isolates and their sensitivities (IC_50_) to ACV or to UL29 siRNA treatment.

HSV-1 Strain	Sample No.	IC_50_ ACV (nM) ^1^	IC_50,_ UL29 Swarm (nM) ^1^
HSV-1 (17+)	-	349.7 ^2^	0.8
HSV-1 (F)	-	803.3	0.7
HSV-1 (Δ305)	-	25468.8 ^2^	1.0
F-14g	H12114	1425.8 ^2^	1.4
F-15	H12115	488.2	1.0
F-17	H12117	995.0 ^2^	2.5
F-18g	H12118	526.1 ^2^	1.1
F-19	H12119	921.1	1.1
M-15	H1215	784.9 ^2^	2.3
M-10	H15110	1023.2	1.9
F-12	H15112	1016.1	0.9
F-15g	H15115	895.1	1.1
F-17/	H15117	527.9	0.9
M-19	H15119	644.9 ^2^	1.7
M-17	H1517	1911.2	3.0
M-19/	H1519	1079.8	0.5
M-01	H151V1	624.0	0.9
M-02	H151V2	559.3	0.9
M-03	H151V3	575.1	0.5
M-04	H151V4	349.7	1.0
Average of circulating strains		844.0	1.3
Standard deviation of circulating strains		379.1	0.7

^1^ The IC_50_ values were determined in Vero cells and are expressed as nM values. The infection was done 4 h post treatment, using 100 plaque forming units/well of each virus. ^2^ ACV sensitivity of the strain according to Bowen et al., 2019 [9].

**Table 3:** Maximal inhibition of viral shedding (I_max_) in response to UL29 siRNA treatment and sequence similarity with the UL29 siRNA swarm target.

Strain	I_max_, UL29 Swarm (%) ^1^	Sequence Similarity (%) ^2^	Differences per 100 Nucleotides ^2^
HSV-1 (17+)	99.85	100.0	0.00
HSV-1 (F)	99.79	99.85	0.15
HSV-1 (Δ305)	96.75	99.21	0.79
F-14g	97.34	99.85	0.15
F-15	98.68	99.84	0.16
F-17	97.95	99.70	0.31
F-18g	99.81	98.93	1.08
F-19	99.22	99.85	0.15
M-15	100.00	99.85	0.15
M-10	100.00	99.85	0.15
F-12	98.23	99.85	0.15
F-15g	100.00	99.85	0.15
F-17/	99.78	99.85	0.15
M-19	99.89	99.85	0.15
M-17	100.00	99.85	0.15
M-19/	98.69	100.0	0
M-01	100.00	99.84	0.16
M-02	99.81	99.69	0.31
M-03	100.00	99.84	0.16
M-04	100.00	99.85	0.15

^1^ Determined in Vero cells and expressed as reduction of viral production (%). The infection was done 4 h post treatment, using 100 plaque forming units/well of each virus. ^2^ In comparison to the UL29 siRNA swarm target: Nucleotides 59,954–59,302 according to the Genbank accession JN555585.1 [13]. An existing Genbank sequence was used for alignments when available.

## Supplementary Materials

The following are available online at https://www.mdpi.com/1999-4915/12/12/1434/s1, Figure S1: Viability of Vero cells after UL29 siRNA swarm treatment. Figure S2: Comparison of inhibition of viral shedding with specific and nonspecific siRNA swarm treatment in Vero cells using a reference strain (HSV-1 (17+)) and an ACV-resistant virus (HSV-1 (Δ305)). Figure S3: UL29 siRNA swarm potency or efficacy do not correlate with RNAi target sequence similarities.
