# Supplementary material for: Herpes Simplex Virus Type 1 Clinical Isolates Respond to UL29-Targeted siRNA Swarm Treatment Independent of Their Acyclovir Sensitivity
Source: Viruses. 2020 Dec 13;12(12):1434. doi: 10.3390/v12121434 (PMC7764767; doi:10.3390/v12121434)
Supplement: Supplementary file 1 [file viruses-12-01434-s001.zip › Kalke_et_al_Figure_S1.pdf]

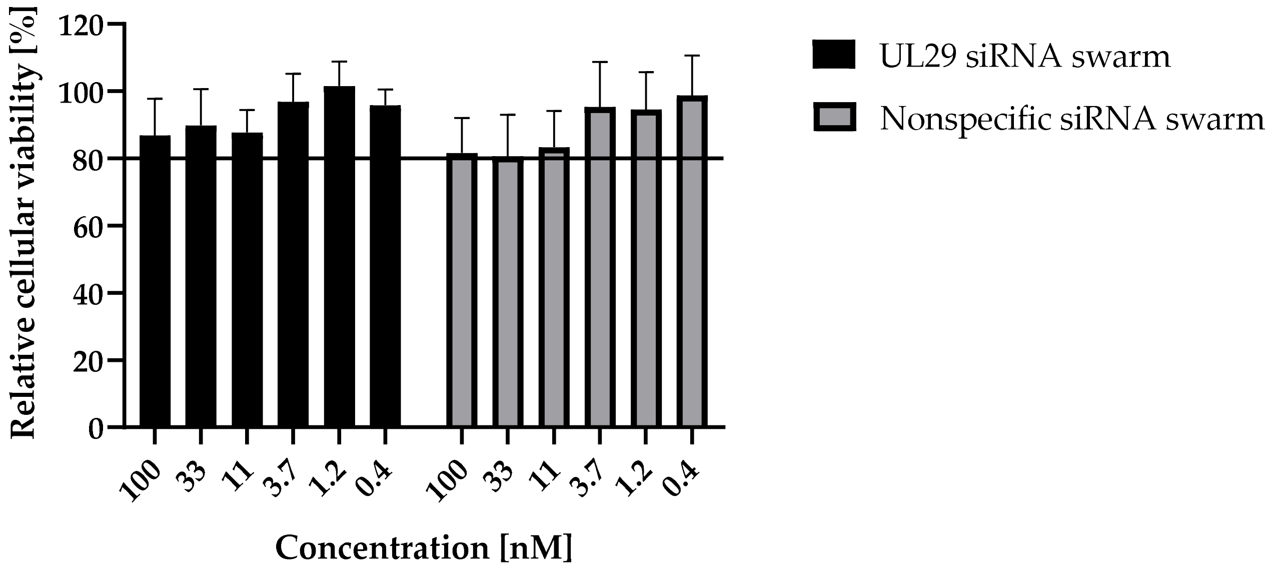


**Supplementary Figure S1 – Viability of Vero cells after UL29 siRNA swarm treatment.** Vero cells were transfected with either UL29 siRNA swam or nonspecific siRNA swarm with 0.4 to 100 nM. Two days post infection, the cellular viability was determined using a luminescent cell viability assay, CellTiter Glo (G7570, Promega, Madison, WI). The derived viability data is shown as relative viability % versus water transfection. The vertical line represents 80% relative viability, which is considered good tolerability. The nonspecific, bacterial *lacI* gene- derived siRNA swarm is previously described in Levanova et al. (2020), ref. [15].
