# Supplementary material for: Herpes Simplex Virus Type 1 Clinical Isolates Respond to UL29-Targeted siRNA Swarm Treatment Independent of Their Acyclovir Sensitivity
Source: Viruses. 2020 Dec 13;12(12):1434. doi: 10.3390/v12121434 (PMC7764767; doi:10.3390/v12121434)
Supplement: Supplementary file 1 [file viruses-12-01434-s001.zip › Kalke_et_al_Figure_S2.pdf]

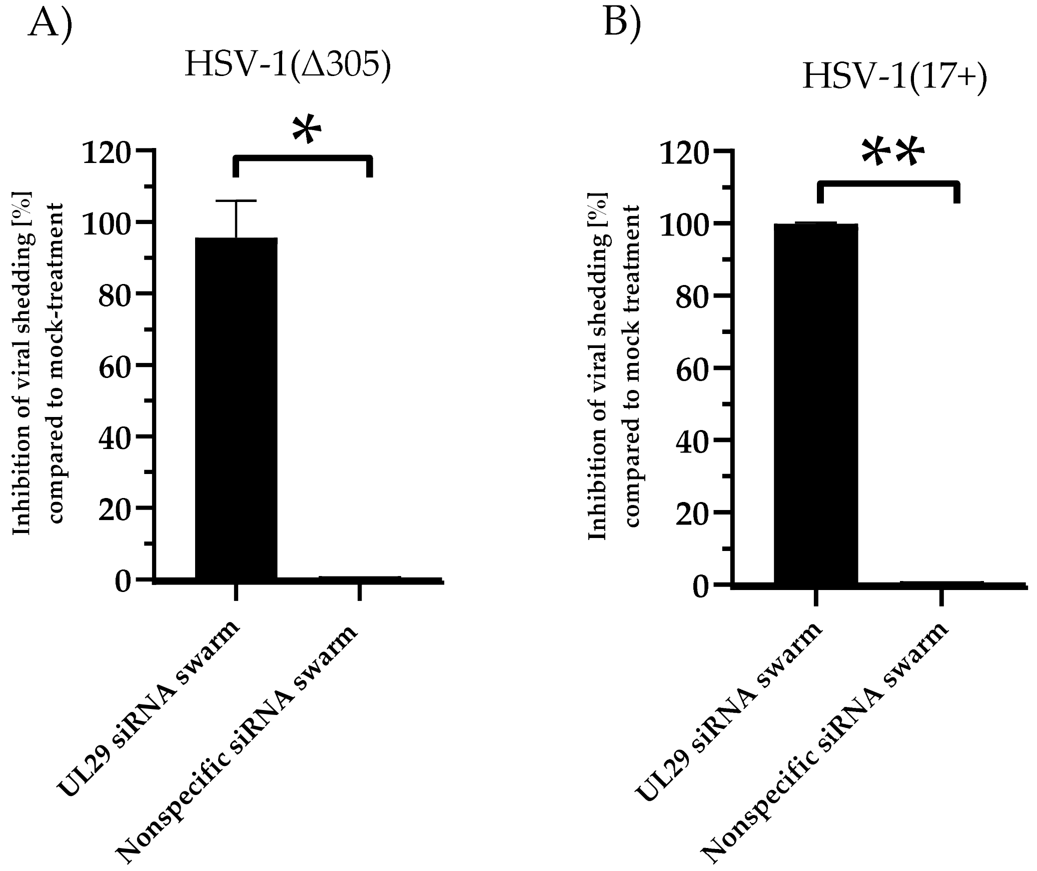

Supplementary Figure S2 – Comparison of inhibition of viral shedding with specific and nonspecific siRNA swarm treatment in Vero cells using a reference strain (HSV-1(17+)) and an ACV resistant virus (HSV-1(Δ305)). Four hours post treatment (hpt) with 100 nM of either specific anti-HSV UL29-siRNA swarm or nonspecific siRNA swarm, the cells were infected with 100 pfu/well of **(A)** HSV-1(Δ305) or **(B)** HSV-1(17+). The experiment was conducted in 96-well plates. Three days post infection (72 hpt), the treated and infected cells were quantified for viral shedding by plaque titration. The inhibition of viral shedding is shown against that of water transfected (mock treated) cells. The data is from two separate experiments (N=4-14). The columns represent the mean and the whiskers the standard deviation. The mean value of inhibition of the nonspecific treatments was negative for both viruses (-168% for HSV-1(17+) and -50% for HSV-1(Δ305)) in comparison to water-treated cells, and for this reason not shown in the graph. The statistical difference between the inhibition with specific and nonspecific siRNA swarms is indicated with asterisks (* $p<0.05$, ** $p<0.01$). The nonspecific siRNA swarm is previously described in Levanova et al. (2020), ref. [15].
