# Supplementary material for: Herpes Simplex Virus Type 1 Clinical Isolates Respond to UL29-Targeted siRNA Swarm Treatment Independent of Their Acyclovir Sensitivity
Source: Viruses. 2020 Dec 13;12(12):1434. doi: 10.3390/v12121434 (PMC7764767; doi:10.3390/v12121434)
Supplement: Supplementary file 1 [file viruses-12-01434-s001.zip › Kalke_et_al_Figure_S3.pdf]

A)

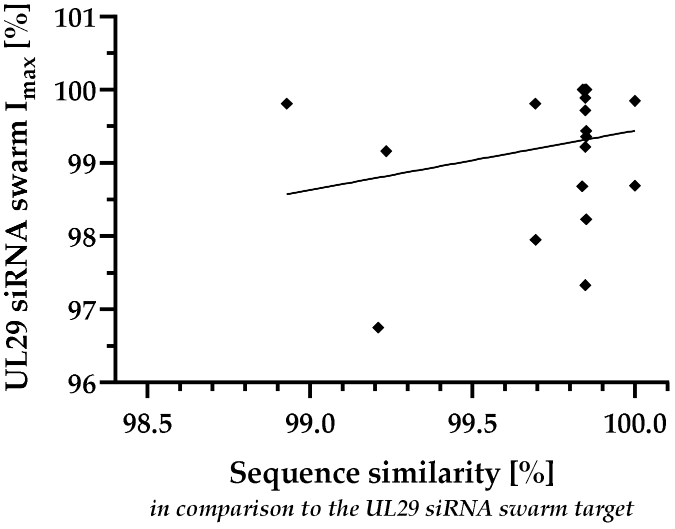

B)

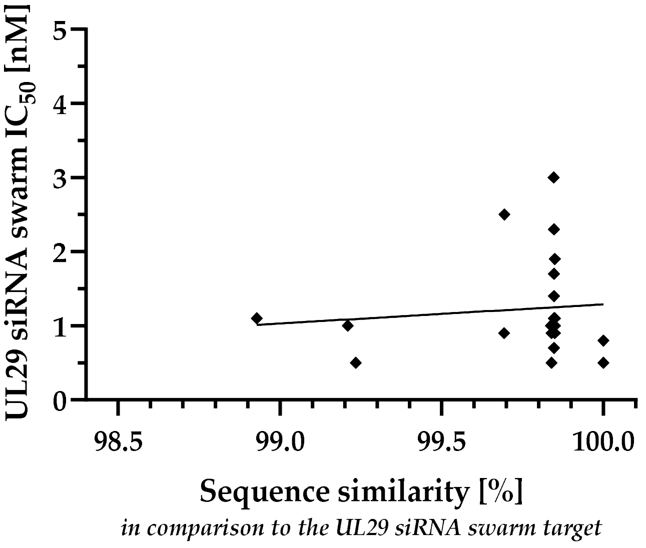

Supplementary Figure S3 – UL29 siRNA swarm potency or efficacy do not correlate with RNAi target sequence similarities. (**A**) Correlation between the UL29 siRNA target sequence similarity and maximal inhibitory efficacy (I_{max}) of UL29 siRNA swarm against the HSV-1 strains. The Spearman's correlation coefficient value (r) for the data set is -0.1566. The correlation was non-significant (p=0.4979). (**B**) Correlation between the UL29 siRNA target sequence similarity and sensitivity to UL29 siRNA swarm. The Spearman's correlation coefficient value for the data set is 0.09715 and the correlation was non-significant (p=0.6753).
